# Yellow Fever—Experts and Things

**Published:** 1897-11

**Authors:** 


### EDITORIAL DEPARTMENT.

F. E. DANIEL, M. D., Editor.
S. E. HUDSON, M. D., Managing Editor.
A. J. SMITH. M. D., Galveston, Associate Editor.

Published Monthly at Austin, Texas, by Drs. Daniel and Hudson. Subscription price $2.00 a year in advance.

Eastern Representatives: Messrs. Monihan & Fairchild, St. Paul Building, 220 Broadway, New York City.

Official organ of the West Texas Medical Association, the Houston District Medical Association, the Austin District Medical Society, the Brazos Valley Medical Association, the Galveston County Medical Society, and several others.

### YELLOW FEVER—EXPERTS AND THINGS.

DR. GUITERAS.—This gentleman has acquired a considerable notoriety in Texas in connection with yellow fever, and in our opinion censure has been unjustly heaped upon him. We feel a sense of shame and humiliation that he should have received at the hands of the medical profession of Galveston so little consideration, if, indeed, the action of a large element of the profession at that place subsequent to the doctor's visit were not indeed an indignity. It is needless to go into details; every reader of this will understand what is alluded to. While we are not particularly partial to the Marine Hospital Service, and would not be thought an apologist for anything that service may do, still we recognize in Dr. Guiteras a gentleman of international reputation and distinction, and as such he is entitled to the respect of his brethren of the medical profession. Dr. Guiteras is but human, and of course is not infallible. If he were in error in his diagnosis—he erred honestly, and it is a shame upon our medical brethren—a scandal upon the profession to have cast at him such insinuations as that he went to Galveston in the interest of New Orleans trade. The writer does not believe that there is money enough in Texas to buy Dr. Guiteras: he is an honorable physician, and as such should be recognized; he is above suspicion. He impressed our State health officer as being a painstaking, exceedingly cautious investigator, and a conscientious student of yellow fever; well informed upon every phase of the subject. We did not have the honor of meeting him, but we have confidence both in his integrity aud his ability.

Our faith in his ability is based upon the fact that he has had abundant opportunity to study, and has studied the disease in its native haunts, and under circumstances enjoyed by few.

It is not generally known, and it affords us pleasure to state, for the benefit of those who are disposed to sneer at what they call his pretensions, that he was one of three distinguished pathologists selected by Congress to investigate yellow fever in the tropics.

The three gentlemen—selected because of their well known ability as pathologists and microscopists, were Drs. George M. Sternberg, now Surgeon-General U. S. Army; Prof. Stanford E. Chaille, the venerable dean of Tulane—and Prof. Jno. Guiteras, our late distinguished visitor,—Professor of Pathology in the University of Pennsylvania. He is a native of Cuba; was raised in Havana. The report of this Commission is well known. Dr. Rudolph Matas, of New Orleans, was Secretary of the Commission. This service alone entitles Dr. Guiteras to be recognized as an expert.

There is a little unwritten history connected with the visit of Dr. Guiteras to Galveston, which we feel inclined to ventilate for the purpose of throwing some light upon the insistance of the Surgeon-General of the Marine Hospital Service that there was yellow fever in Galveston long anterior to the coming of Dr. Guiteras; and as showing the reason why Dr. Guiteras was sent there. It is this: Early in September—just after the first case had occurred in New Orleans—or about the time of the occurrence of the seven suspicious cases there, which Health Officer Fisher, of Galveston, so bitterly denounced what he called "Swearingen's quarantine," for not operating against;—said it was "a *perfect farce*"—his reason for saying so being that Swearingen did not quarantine against New Orleans on the occurrence of these suspicious cases,—there occurred a case of sickness in the Seeley Hospital at Galveston—and a death, under circumstances which were more than suspicious. It was so suspicious that an autopsy was thought necessary. It was made, and we were informed some weeks afterwards,—that Prof. Allen J. Smith, the pathologist of the medical college and hospital, gave it as his opinion that the case was yellow fever. Dr. Smith was so impressed with the conviction that it was yellow fever that he isolated himself for a week, and took all the precautions he would have taken against a known and positive case of yellow fever. Knowledge of this case was suppressed. But it got to Surgeon-General Wyman, of the Marine Hospital Service, in some way,—and he telegraphed to Dr. Swearingen more than once: "I have information by wire—seemingly perfectly reliable, that yellow fever exists in Galveston." Dr. Swearingen had to reply that he had no knowledge of the existence of a single case there. The Surgeon-General was not satisfied and was never convinced. Hence his insistance on sending a representative there to investigate.

The State Health Officer never heard of the occurrence of this case until thirteen days after the death and autopsy. When he he did get information of it at the hands of a friend, in a personal letter, he sent a telegram to Dr. Fisher, the health officer at Galveston, asking if a death had occurred at the Sealy Hos pital on such a day; if there was any autopsy made; if so, what was the result. To this inquiry Dr. Fisher replied that there had been a death on the day stated; that an autopsy had been made and that Prof. Smith thought it was yellow fever; *but* that, as thirteen days had elapsed and no results had followed, it had been concluded that it was not yellow fever; or words to that effect.

The above is submitted without comment,—further than to say that upon the occurrence of a "suspicious case"—believed by competent authority to be yellow fever—it is the duty of a health officer to give the people the benefit of the doubt. In this case it was clearly the duty of the local health officer to report the existence of a suspicious case to the State Health Officer. Why did Dr. Fisher suppress all knowledge of this case? His conduct with reference to it contrasts strikingly with the great concern he manifested later, lest even Dr. Guiteras should bring in the germs. Dr. Truehart said at a council meeting "that any physician who would conceal the presence of a case of yellow fever, ought to be tarred and feathered." What should be done to a health officer who would conceal the presence of a more than suspicious case?

It does seem to make a great deal of difference whose ox is gored. Dr. Fisher was very indignant that the State Health Officer did not clap on a quarantine against New Orleans when several suspicious cases were reported as occurring on one block, and denounced the State Quarantine as "a perfect farce." We may state here that the Louisiana State Board of Health had wired the Texas Quarantine Department that they had the cases isolated and measures to prevent a spread of the disease, in case it should prove to be yellow fever, had been promptly instituted. To have quarantined against suspicious cases in the face of such assurances would have been discourteous to the Louisiana Board, to say the least; a want of confidence in their integrity. *Now*, when "suspicious cases" (?) occur in Galveston; nay, when the highest authority in both the National and the State service pronounce it yellow fever, and quarantine is declared against Galveston, there are none so loud in their protests as Dr. Fisher.

AND, BY THE BYE—I see that inquiry has been made—rather sneeringly—by what right or title, Dr. Guiteras poses as expert in yellow fever, which inquiry, we are pleased to see, that Dr. Allen J. Smith answers in the *Galveston News*, showing Dr. Guiteras has claims to be so regarded, and claims to the confidence of his superior officer. It would be pertinent to inquire, in this connection, where did so many native "experts" come from? Where, for instance, did Dr. R. H. Harrison, Sr., of Columbus, Texas, graduate in yellow fever? Who conferred upon him the degree "Expert"? By what warrant does *he* go to Galveston to investigate? To whom is *he* expected to report, and, finally, what does *his* opinion amount to any way? We see that he has been making speeches before the Galveston council and getting himself interviewed, and his dictum seems to be taken at par,—without any discount,—*because* he says there is no yellow fever in Galveston. We are here reminded of the old preacher who begged to differ with Brother Paul about something Paul had said in his epistles to the—Marines—no, the Corinthians or the Ephesians.

THEN THERE IS THE FIASCO of ten or a dozen local health authorities—county physicians—treated to a free ride to Galveston and back on a tour of inspection, not one of whom ever saw a case or yellow fever. These gentlemen—by the bye—the names of some of them are entirely new to us,—unfamiliar,—and we have published this JOURNAL nearly thirteen years—look the ground over, after the battle, and certify that there has been no yellow fever in Galveston and Houston, and that it is all a mistake, and, in effect, say that Swearingen, Guiteras, West and Truehart, and Turner (of Houston),—all to the contrary—don't know what they are talking about; that's all. "We, the people," said the four tailors in Thread-needle-steel, "resolve," etc. This reminds us of the boy who claimed to have killed one hundred squirrels on one tree, and "if you don't believe it," said he, "I can show you the stump of the tree." Upon what data do these gentlemen base their assertion, that there *has not been* any yellow fever in those cities?

