# That Case at Galveston

**Published:** 1897-12

**Authors:** 


THAT CASE AT GALVESTON. A CORRECTION.—Shortly after the November number of the JOURNAL was out my attention was called by State Health Officer Swearingen to the fact that the statement on page 241, that in reply to a telegram from him about a case said to have been yellow fever, "Dr. Fisher replied that an autopsy had been made and that Prof. Smith thought it was yellow fever," was incorrect. I immediately wrote Dr. Fisher stating that I had written from recollection of a conversation with Dr. Swearingen in which I got the contents of a *letter* he read to me and the *telegram* mixed; that it was an error on my part which I much regretted, and that in the next issue I would correct it. I now give the telegraphic correspondence *verbatim*, having the originals before me. Dr. Swearingen on September 16th, received a private letter from a responsible physician in Galveston conveying the information that some days prior to that writing, one Guadalupe Torres had died in Seely hospital; that an autopsy was made and that Prof. Allen J. Smith pronounced the case yellow fever, and immediately isolated himself, fearing he might have the fever and spread it. On receipt of this letter Dr. Swearingen sent the following telegram to Dr. Fisher, City Physician, Galveston:

AUSTIN, TEX., Sep. 17, 1897.

*Dr. W. C. Fisher, Galveston, Texas.*

"What of the Mexican, Guadalupe Torres, who recently died at City Hospital? Did Dr. Smith or any one hold autopsy? If yes, what results?" R. M. SWEARINGEN.

To this telegram he received the following reply:

GALVESTON, Sep. 17, 1897.

*Dr. R. M. Swearingen, Austin, Texas.*

From clinical history, personal history as to locality, catarrhal jaundice. Now thirteen days since inception of disease and no developments suspicious." Signed,

F. K. FISHER.

It will be seen that Dr. Fisher did not reply specifically to the inquiry as to autopsy by Prof. Smith—omitted it, or *evaded* it. If he had answered it he *would have had to say* "an autopsy was made, and Prof. Smith thought it was yellow fever." So I only put into his mouth the words he would have had to use if he had not—purposely or otherwise—failed to answer a direct question. So I cannot see that I am far from the fact, after all. Dr. Fisher has a statement in this issue.

---

HAMILTON, of the *Journal American Medical Association* gets off a good one on Supervising Surgeon-General Marine Hospital Service, whom he once designated as "one Wyman." He says (*Journal American Medical Association*) that at the recent meeting of the American Public Health Association when the bill to create a department of public health was being considered:

"A feeble protest against this reference was made at the meeting by the representative of the Marine-Hospital Service on the ground that it was inappropriate for an Association composed in part of Canadian and Mexican members to unite in a proposition concerning only the United States, but the *individual protesting* (meaning "one Wyman") was reminded that he himself had endeavored, only a year ago, at Buffalo, to obtain from the same tripartite body the passage of a resolution recommending that the U. S. Marine Hospital service should have its powers so enlarged as to constitute *it* the National Board of Health of the United States, and that he had complained in an official report because, as he alleged, a member of the Executive Committee had taken it upon himself to forestall its adoption. The partisan spirit exhibited in this querulous objection is additional reason why a Department of Public Health should be a Commissioner's Bureau independent of any other existing organization, with intimate relations with the State Boards of Health—harmonizing, supplementing and co-operating with them instead of subverting and antagonizing or ignoring them. A health department of this kind is the great immediate National need and the Congress should no longer delay to enact it."

